# Rapid hepatic clearance of full length CCN-2/CTGF: a putative role for LRP1-mediated endocytosis

**DOI:** 10.1007/s12079-016-0354-6

**Published:** 2016-09-19

**Authors:** K. G. F. Gerritsen, N. Bovenschen, T. Q. Nguyen, D. Sprengers, M. P. Koeners, A. N. van Koppen, J. A. Joles, R. Goldschmeding, R. J. Kok

**Affiliations:** 1 Department of Pathology, University Medical Center Utrecht, Heidelberglaan 100, 3584 CX Utrecht, The Netherlands; 2 Department of Nephrology and Hypertension, University Medical Center Utrecht, Utrecht, The Netherlands; 3 Department of Gastroenterology and Hepatology, Erasmus MC University Medical Center, Rotterdam, The Netherlands; 4 Department of Pharmaceutics, Utrecht Institute for Pharmaceutical Sciences, Utrecht, The Netherlands

**Keywords:** Biomarker, CCN-2, CTGF, Hepatic clearance, LRP1

## Abstract

CCN-2 (connective tissue growth factor; CTGF) is a key factor in fibrosis. Plasma CCN-2 has biomarker potential in numerous fibrotic disorders, but it is unknown which pathophysiological factors determine plasma CCN-2 levels. The proteolytic amino-terminal fragment of CCN-2 is primarily eliminated by the kidney. Here, we investigated elimination and distribution profiles of full length CCN-2 by intravenous administration of recombinant CCN-2 to rodents. After bolus injection in mice, we observed a large initial distribution volume (454 mL/kg) and a fast initial clearance (120 mL/kg/min). Immunosorbent assay and immunostaining showed that CCN-2 distributed mainly to the liver and was taken up by hepatocytes. Steady state clearance in rats, determined by continuous infusion of CCN-2, was fast (45 mL/kg/min). Renal CCN-2 clearance, determined by arterial and renal vein sampling, accounted for only 12 % of total clearance. Co-infusion of CCN-2 with receptor-associated protein (RAP), an antagonist of LDL-receptor family proteins, showed that RAP prolonged CCN-2 half-life and completely prevented CCN-2 internalization by hepatocytes. This suggests that hepatic uptake of CCN-2 is mediated by a RAP-sensitive mechanism most likely involving LRP1, a member of the LDL-receptor family involved in hepatic clearance of various plasma proteins. Surface plasmon resonance binding studies confirmed that CCN-2 is an LRP1 ligand. Co-infusion of CCN-2 with an excess of the heparan sulphate-binding protamine lowered the large initial distribution volume of CCN-2 by 88 % and reduced interstitial staining of CCN-2, suggesting binding of CCN-2 to heparan sulphate proteoglycans (HSPGs). Protamine did not affect clearance rate, indicating that RAP-sensitive clearance of CCN-2 is HSPG independent. In conclusion, unlike its amino-terminal fragment which is cleared by the kidney, full length CCN-2 is primarily eliminated by the liver via a fast RAP-sensitive, probably LRP1-dependent pathway.

## Introduction

CTGF/CYR61/NOV −2 (CCN-2, also known as connective tissue growth factor or CTGF) is a key factor in the pathogenesis of organ fibrosis. Circulating CCN-2 correlates with fibrogenic activity in various chronic disorders and might be useful as a non-invasive marker for monitoring fibrosis, including liver fibrosis (Dendooven et al. 2011; Gressner et al. 2013; Guo-Qiu et al. 2010; Bauer et al. 2012; Honsawek et al. 2013; Colak et al. 2012). The CCN-2 molecule is a 349–amino acid polypeptide of 36–38 kDa which consists of four distinct cysteine-rich interaction domains. Between domain 2 and 3 the protein contains a cysteine-free hinge region, which is susceptible to proteolytic cleavage generating N- and C-terminal fragments of similar molecular weight. The N-fragment is the predominant CCN-2 molecule in plasma in healthy subjects and chronic kidney disease patients, while full length CCN-2 is hardly detectable by available techniques (Dendooven et al. 2011). In chronic liver disease, however, elevated circulating levels of the full length protein were reported (Gressner et al. 2006; Guo-Qiu et al. 2010). Only sparse data are available on circulating C-fragment levels and reported levels were lower than those of the N-fragment and the full length molecule (Weitz and Usinger 2003; Dziadzio et al. 2005).

Circulating CCN-2 levels are determined by tissue production, release into the circulation, proteolytic cleavage, cell- and matrix interaction and elimination processes. CCN-2 tissue expression is generally increased in chronic fibrotic disorders (Dendooven et al. 2011). However, plasma CCN-2 profiles differ between various disorders perhaps because of different pharmacokinetic profiles of the CCN-2 full-length protein and its fragments and interference with different elimination routes depending on which organ is affected. Previously, we demonstrated that the N-fragment of CCN-2 is primarily eliminated by the kidney resulting in accumulation of circulating N-fragment in chronic kidney disease (CKD) (Gerritsen et al. 2012). The high levels of full length CCN-2 in chronic liver disease and negligible levels in CKD suggest involvement of the liver in clearance of the whole protein. Gao et al. reported in vitro binding of CCN-2 via domain 3 to low density lipoprotein receptor-related protein 1 (LRP1) (Gao and Brigstock 2003), a multifunctional endocytic receptor highly expressed in liver and known to be involved in cellular uptake and subsequent degradation of various proteins (Lillis et al. 2008). Liver cirrhosis is associated with decreased expression of LRP1 (Hollestelle et al. 2004). Low levels of full length CCN-2 in healthy subjects might also be explained by binding to cell surface or matrix. Previous reports show in vitro binding of CCN-2 to heparan sulphate proteoglycans (HSPGs) (Gao and Brigstock 2004). Since HSPGs are widely distributed, full length CCN-2 released into the circulation might be rapidly captured by HSPGs and removed from plasma.

To investigate elimination and distribution profile of full length CCN-2, we administered recombinant human full length CCN-2 intravenously to rodents. In addition, we examined the involvement of LRP1 and HSPGs in clearance and distribution of full length CCN-2 by co-infusion of competing ligands.

## Materials and methods

### Proteins

Recombinant CCN-2 s and anti- CCN-2 antibodies were supplied by FibroGen Inc. (San Francisco, CA). The recombinant human CCN-2 s were produced in a baculovirus expression system in Chinese Hamster Ovary cell lines cultured in hollow-fiber fermentors. The proteins were purified by CCN-2-affinity and cation exchange chromatography. Recombinant full length CCN-2 comprised all four domains, the N-terminal fragment comprised domain 1 and 2 and C-terminal fragment comprised domain 3 and 4. Purified placenta-derived LRP1 was a kind gift by Dr. S. K. Moestrup (University of Aarhus, Aarhus, Denmark) (Moestrup and Gliemann 1991). The bacterial vector encoding glutathioneS-transferase-fused receptor-associated protein was kindly provided by Dr. J. Kuiper (Leiden University, Leiden, The Netherlands). Glutathione S-transferase-fused receptor-associated protein (GST-RAP) was expressed in *E. coli* strain DH5α and purified by glutathione-Sepharose (Herz et al. 1991).

### A*nimal experiments*

Pharmacokinetic studies were performed in mice and rats. All experiments were approved by the Animal Ethical Committee of the University of Utrecht and performed in accordance with national guidelines for the care and handling of animals. C57Bl/6 J mice (12 weeks old; Harlan, Horst, The Netherlands) received recombinant human CCN-2 by tail vein injection (25 pmol/g bodyweight, single dose) (Gerritsen et al. 2010). A blood sample was drawn by cheek puncture to determine the initial plasma concentration in each individual mouse. To evaluate the plasma disappearance and uptake in organs, mice were sacrificed at various time points (*n* = 2 per time point). Kidneys, liver, lungs, heart and spleen were harvested, weighed, and processed for ELISA or immunohistochemistry, as described below. To evaluate the effects of blocking of LRP1 or HSPGs two separate groups of mice received either GST-RAP (0.7 nmol/g bodyweight, *n* = 8) or protamine hydrochloride (ICN Biomedicals, Zoetermeer, The Netherlands) (5 nmol/g bodyweight, *n* = 7), respectively, or vehicle (*n* = 6 for both experiments) by tail vein injection 1 min before CCN-2 injection in the other tail vein (25 pmol/g bodyweight). Previous reports showed that these amounts of RAP and protamine were adequate to saturate LRP1 and to block HSPGs sufficiently to influence the pharmacokinetics of its ligands, respectively (Saenko et al. 1999; Gerritsen et al. 2010; Narita et al. 1995).

Studies in rats were performed to quantify renal and non-renal CCN-2 clearance. Male Wistar-Kyoto rats (15–17 weeks old; Harlan) were anaesthetized and instrumented as described (Gerritsen et al. 2010). After a 45 min stabilization period, infusion of full length CCN-2 was started at 54 pmol/kg/min, based on pilot clearance studies. Inulin (Inutest, Fresenius Pharma, Linz, Austria) and PAH (Sigma, St Louis, MO) were infused for renal clearance measurements. Urine was collected at 15 min intervals. 90 Min after the start of CCN-2 infusion two arterial blood samples were taken at an interval of 30 min. After a short equilibration period a third arterial blood sample was taken simultaneously with puncture of the left renal vein to determine renal extraction and clearance. Inulin and PAH were measured as described (Verseput et al. 1997).

### CCN-2 enzyme-linked immunosorbent assay

Recombinant human CCN-2 levels in plasma, urine, and tissue homogenates were determined by sandwich enzyme-linked immunosorbent assay (ELISA), detecting both full length CCN-2 and the N-terminal fragment. A human CCN-2 specific assay was used, that does not cross-react with rodent CCN-2, as described (Gerritsen et al. 2010; Gerritsen et al. 2015). Frozen renal tissue (stored at −80 °C) was homogenized in lysis buffer (20 mM Tris (Roche, Mannheim, Germany), 150 mM NaCl (Merck, Darmstadt, Germany), 1 % Triton X-100, 10 % glycerol, 1 mM EDTA (Riedel-de Haen, Seelze, Germany), 0.1 % SDS (Research Organics, Cleveland, OH), 1 mM EGTA, 0.5 % sodium deoxycholate, 50 mM NaF, 2 mM Na-orthovanadate (Sigma), pH 7.4) containing 5 % Protease Inhibitor Cocktail (Sigma). Microtiter plates (Maxisorb; Nunc, Roskilde, Denmark) were coated with capture anti- CCN-2 monoclonal antibody (5 μg/ml; FibroGen). Subsequently, diluted samples and standards (recombinant human CCN-2; FibroGen) were added and incubated with non-cross-blocking human anti- CCN-2 monoclonal antibody conjugated directly to alkaline phosphatase (0.5 μg/ml; FibroGen). Para-nitrophenylphosphate (Sigma) was used as substrate for the colorimetric reaction. Assay sensitivity (lower limit of detection) was 20 pmol/L. Intra- and interassay coefficients of variation were below 10 %.

### Immunofluorescence

Immunostaining for CCN-2 was performed on 3 μm formalin-fixed paraffin-embedded tissue sections. After deparaffinization, antigen retrieval was performed by predigestion with Protease XXIV (0.2 M phosphate; Sigma) followed by blocking of endogenous peroxidase activity (1 % H_2_O_2_ in phosphate/citrate buffer). Sections were incubated with CCN-2-specific human monoclonal antibody (FibroGen) (24 μg/ml in PBS/1 % BSA) for 1 h followed by incubation with rabbit anti-human IgG (Dako, Glostrup, Denmark) (1:100, 30 min). Amplification was performed with Powervision poly-peroxidase goat anti-rabbit IgG (Klinipath, Duiven, The Netherlands) (30 min) and FITC Tyramide Amplification Reagent (PerkinElmer, Boston, MA) (1:50, 10 min). TO-PRO 3 Iodide (Molecular Probes, Eugene, OR) was used for nuclear counterstaining. Slides were mounted in Vectashield (Vector Laboratories, Ontario, Canada), and visualized by fluorescence and confocal laser scanning microscopy. For all stainings, incubation with secondary antibody alone served as a negative control.

### Surface Plasmon resonance analysis

Real-time binding experiments were performed on the Biacore T100 (GE Healthcare, Uppsala, Sweden). LRP1 was immobilized on a Series S CM5 sensor-chip surface at 3.4 fmol/mm2. One control flow channel was routinely activated and blocked in the absence of protein. Association of recombinant human CCN-2 and its proteolytic fragments and inhibition by RAP were assessed in 10 mM Hepes (pH 7.4), 150 mM NaCl, 5 mM CaCl2 and 0.005 % surfactant P20 for 2 min, at a flow rate of 20 μl/min at 25 °C. Dissociation was allowed for 2 min in the same buffer flow. Sensor chips were regenerated using EDTA 125 mM, NaCl 0.5 M during at a flow rate of 20 μl/min for 30 s. Proteins were injected until binding equilibrium was reached. Data were corrected for both refractive index changes and were analyzed with BIAcore T100 evaluation software (version 2.01). Affinity constants were determined by steady-state analysis. The inhibitory effect of RAP on the interaction of CCN-2 with LRP1 was assessed by adding RAP (3 μM) to immobilized LRP1 followed by CCN-2 (1 μM). Subsequently, binding of CCN-2 to RAP-saturated LRP1 was compared with that of CCN-2 (1 μM) to LRP1 in the absence of RAP.

### Calculations and statistics

Data are expressed as mean ± SE and were compared with Student’s *t*-test (2-tailed). Pharmacokinetic analysis was performed using a nonlinear two-compartment model (Multifit program, Department of Pharmacokinetics and Drug Delivery, University of Groningen, The Netherlands) (Prakash et al. 2006). Renal clearances of inulin and PAH were calculated by standard formulae. Total CCN-2 clearance was calculated as follows:

$$ \frac{\mathrm{infusion}\ \mathrm{rate}}{\mathrm{arterial}\ \mathrm{concentration}} $$

Renal vein concentration was used to determine the amount of CCN-2 extracted by the kidney and renal CCN-2 clearance was calculated as follows:

$$ \frac{\left(\mathrm{arterial}\ \mathrm{concentration}\times \mathrm{ERPF}\right)-\left(\mathrm{renal}\ \mathrm{vein}\ \mathrm{concentration}\times \left(\mathrm{ERPF}-\overset{\bullet }{\mathrm{V}}\right)\right)}{\mathrm{arterial}\ \mathrm{concentration}\ } $$

where ERPF is the effective renal plasma flow and $\overset{\bullet }{V}$the urinary flow rate.

## Results

### Circulating full length CCN-2 is primarily eliminated by hepatic metabolism

**Fig. 1:**
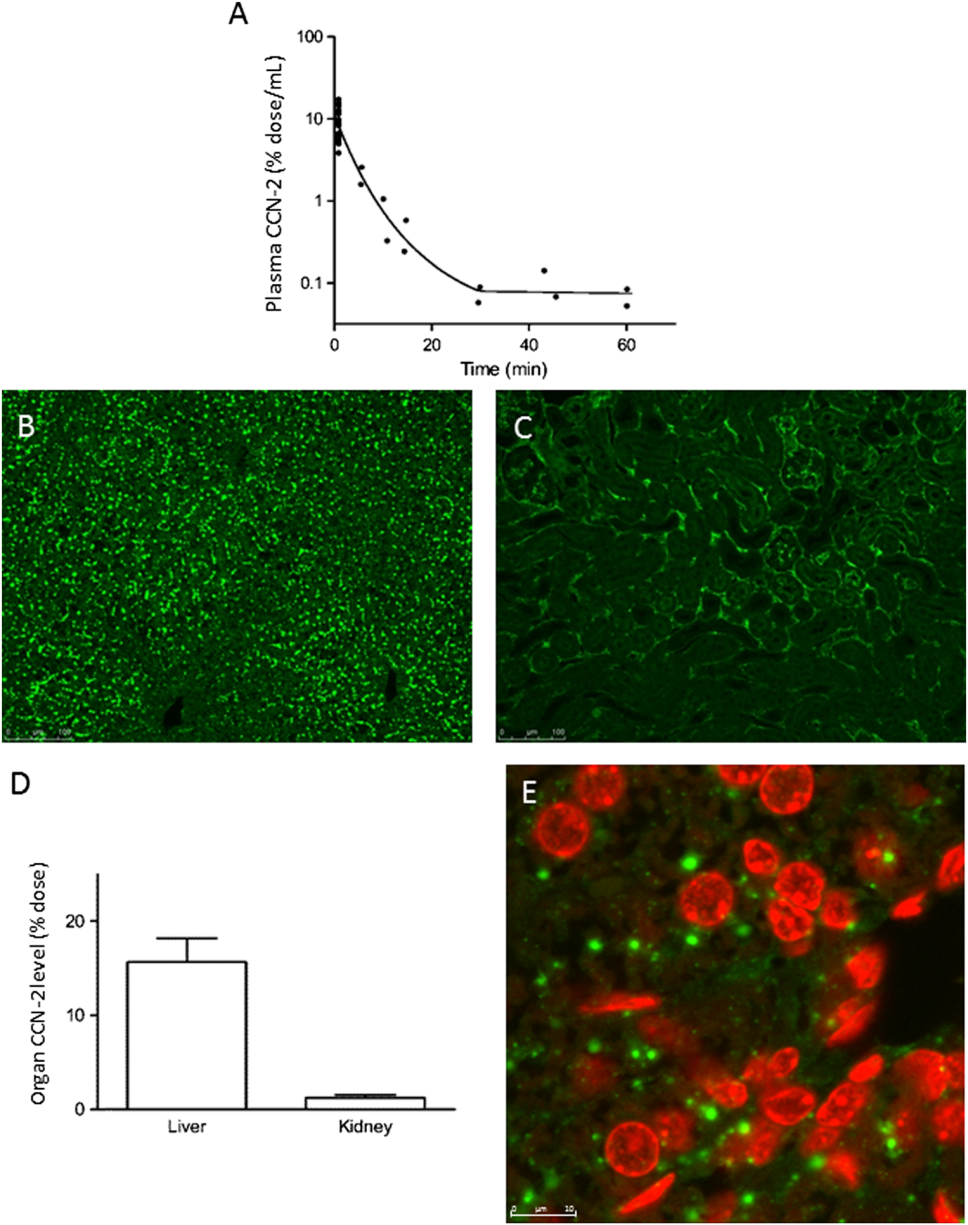
Pharmacokinetic profile after single intravenous dose of recombinant human CCN-2 in mice. **a** Plasma disappearance curve of CCN-2. Plasma concentration expressed as percentage of injected dose per mL plasma. Each dot represents one animal. **b**-**c** Immunofluorescence of liver **b** and kidney **c**, 15 min after CCN-2 administration, by using human anti- CCN-2 (*green*), visualized by fluorescence microscopy. **d** Estimated organ levels expressed as percentage of injected dose of recombinant human CCN-2 5–10 min after intravenous CCN-2 administration in mice (*n* = 8). **e** Immunofluorescence of liver by using human anti- CCN-2 (*green*) and TO-PRO 3 Iodide for nuclear staining (*red*), visualized by confocal laser scanning microscopy

First, we investigated the pharmacokinetic profile of recombinant human CCN-2 that was administered intravenously as a single bolus to healthy mice. Human CCN-2 specific antibodies were used to distinguish recombinant human CCN-2 from endogenous CCN-2. The plasma disappearance curve is shown in Fig. 1a. The initial distribution volume was relatively large for a hydrophilic protein such as CCN-2: 454 ± 47 mL/kg. This value exceeds plasma and extracellular fluid volume suggesting tissue binding of CCN-2. The plasma disappearance rate during the first 15 min was fast with an initial clearance of 120 ± 11 mL/kg/min corresponding to an initial half-life of 2.6 ± 0.02 min. Since plasma levels dropped rapidly to very low levels, no reliable pharmacokinetic data could be obtained after the initial distribution phase. To obtain a more accurate estimate of CCN-2 clearance and to quantify renal and non-renal clearance, additional experiments were conducted with continuous CCN-2 infusion. Because we had to take multiple blood samples, we performed these experiments in anaesthetized rats. After reaching steady state, kinetic parameters were calculated from plasma concentrations and urinary excretion, as summarized in Table 1. Total CCN-2 clearance was derived from steady state plasma concentration and infusion rate. For determination of renal extraction and clearance, blood samples were drawn simultaneously from the renal vein and femoral artery. Total CCN-2 clearance was 45 ± 8 mL/kg/min with renal clearance 5.4 ± 0.8 mL/kg/min, accounting for only ~12 % of total clearance, indicating that the majority of the protein was cleared via a non-renal route.

**Table 1:** Clearance of recombinant human full length CCN-2 in healthy rats

Parameter	*N* = 4
Body weight (g)	309 ± 11
Mean arterial pressure (mmHg)	101 ± 2
GFR (mL/kg/min; inulin)	9.0 ± 0.1
Renal plasma flow (mL/kg/min; PAH)	30 ± 1
Total CCN-2 clearance (mL/kg/min)	45 ± 8
Renal CCN-2 clearance (mL/kg/min)	5.4 ± 0.8

Values are means ± SE. GFR, glomerular filtration rate; PAH, para- aminohippuric acid

To investigate CCN-2 tissue distribution we measured CCN-2 tissue levels and performed immunohistochemistry after single bolus injection in mice. We observed rapid accumulation of CCN-2 in the liver and to a lesser extent in the kidney (Fig. 1b-d). Immunofluorescent staining showed uptake of CCN-2 in intracellular organelles in hepatocytes (Fig. 1e), which was observed only temporarily during the first 15 min after injection suggestive of rapid hepatic metabolism. In the kidney, CCN-2 was filtered and subsequently endocytosed to a large extent by proximal tubular cells, as reported previously (Gerritsen et al. 2010). We found practically no distribution to other tissues including lung, heart or spleen. Thus, in healthy rodents circulating full length CCN-2 is primarily eliminated by the liver and to a lesser extent by the kidney.

### RAP prolongs CCN-2 half-life and prevents CCN-2 internalization by hepatocytes

**Fig. 2:**
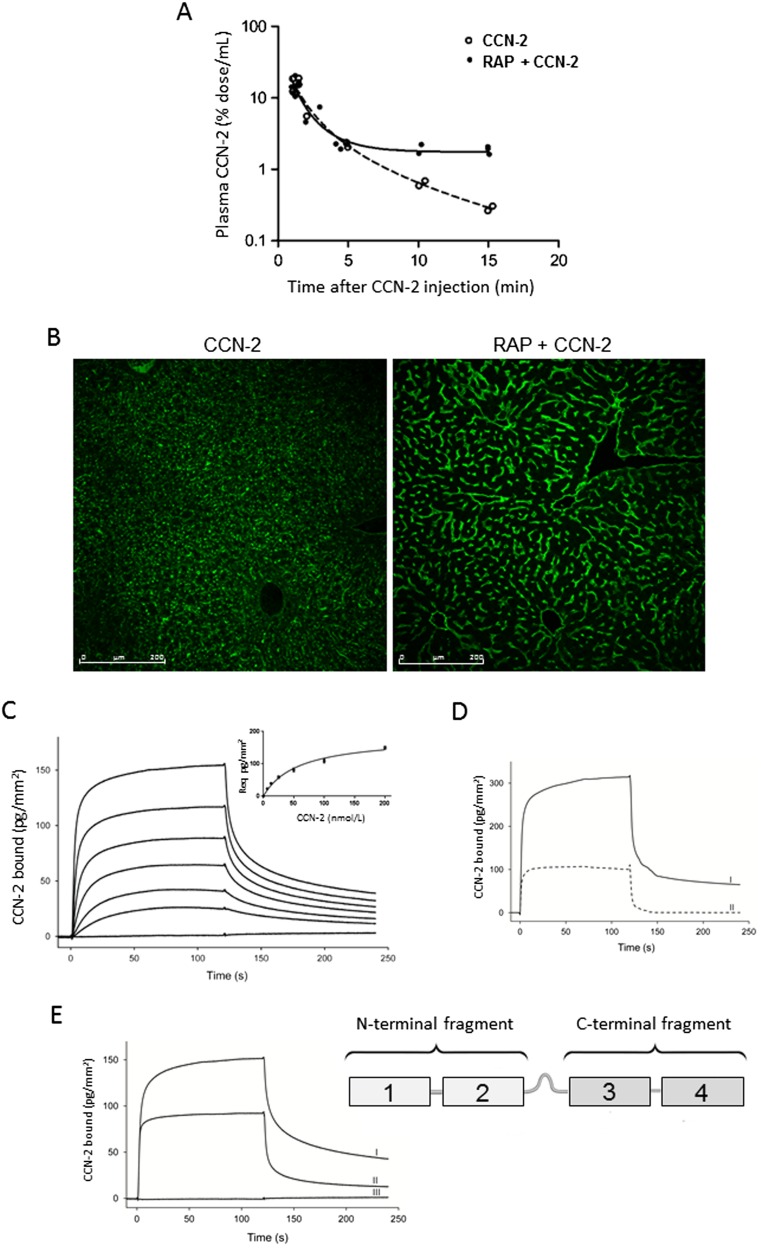
**a**-**b** Pharmacokinetic profile after single intravenous dose of recombinant human CCN-2 preceded by injection of an excess of GST-RAP in mice. **a** Plasma disappearance curve of CCN-2. GST-RAP prior to CCN-2 reduced the elimination rate but not initial distribution. Plasma concentration is expressed as percentage of injected dose per mL plasma. Each dot represents one animal. **b** Immunofluorescence of liver 15 min after CCN-2 administration, by using human anti- CCN-2 (*green*), visualized by confocal laser scanning microscopy. GST-RAP prior to CCN-2 (*right*) reduces uptake in hepatocytes. **c**-**e** Surface plasmon resonance analysis of human CCN-2 and its proteolytic fragments, run over immobilized LRP1. Response is depicted as pg/mm^2^. **c** Dose-dependent response with concentrations of full length CCN-2 of 0, 6.25, 12.5, 25, 50, 100 and 200 nM. **d** Interaction of CCN-2 (1 uM) (I) with LRP1 is partially inhibited by GST-RAP (3 uM) (II). **e** Full length CCN-2 (200 nM) (I) and C-terminal fragment (200 nM) (II), but not N-terminal fragment (200 nM) (III), show interaction with LRP1

We studied the effect of RAP, an antagonist of low density lipoprotein receptor (LDLR) family proteins, on the pharmacokinetic profile of CCN-2. In mice, intravenous injection of an excess of GST-RAP 1 min prior to intravenous bolus injection of CCN-2 reduced elimination rate of CCN-2 after a comparable initial distribution phase (Fig. 2a), resulting in 5-fold higher plasma CCN-2 concentrations at 10–15 min after injection (*p* < 0.001). Immunohistochemistry showed less uptake in intracellular organelles in hepatocytes, suggesting reduced intrahepatic degradation (Fig. 2b). These results suggest that hepatic metabolism of circulating CCN-2 is dependent on its uptake in hepatocytes by an LDLR family member, most likely LRP1. RAP has high affinity for LRP1 (K_*d*_ 5.4 nM) and is known to inhibit binding and endocytosis of all known LRP1 ligands (Williams et al. 1992). Administration of a bolus GST-RAP alone did not result in detectable levels of circulating endogenous full length CCN-2 15 min after injection (data not shown). This indicates that CCN-2 release from tissues is very low in healthy mice.

To confirm that CCN-2 is an LRP1 ligand, we performed surface plasmon resonance analysis with immobilized LRP1. CCN-2 bound to LRP1 in a dose-dependent manner (Fig. 2c). The binding affinity (*K*_*d*_) of this interaction was calculated to be 116 ± 22 nM. Interaction of CCN-2 with LRP1 was at least partially inhibited by RAP (Fig. 2d). Next, interaction of individual proteolytic CCN-2 fragments with LRP1 was analyzed. Interestingly, the C-terminal part of CCN-2 interacted with LRP1 (*K*_*d*_ = 112 ± 7 nM) with similar affinity as did full-length CCN-2. In contrast the N-terminal part of CCN-2 did not show any binding to immobilized LRP1, up to concentrations of 1 uM. These data indicate that LRP1 binds to CCN-2 through LRP binding sites in its C-terminal moiety.

### Blocking of HSPGs reduces CCN-2 distribution volume in vivo

**Fig. 3:**
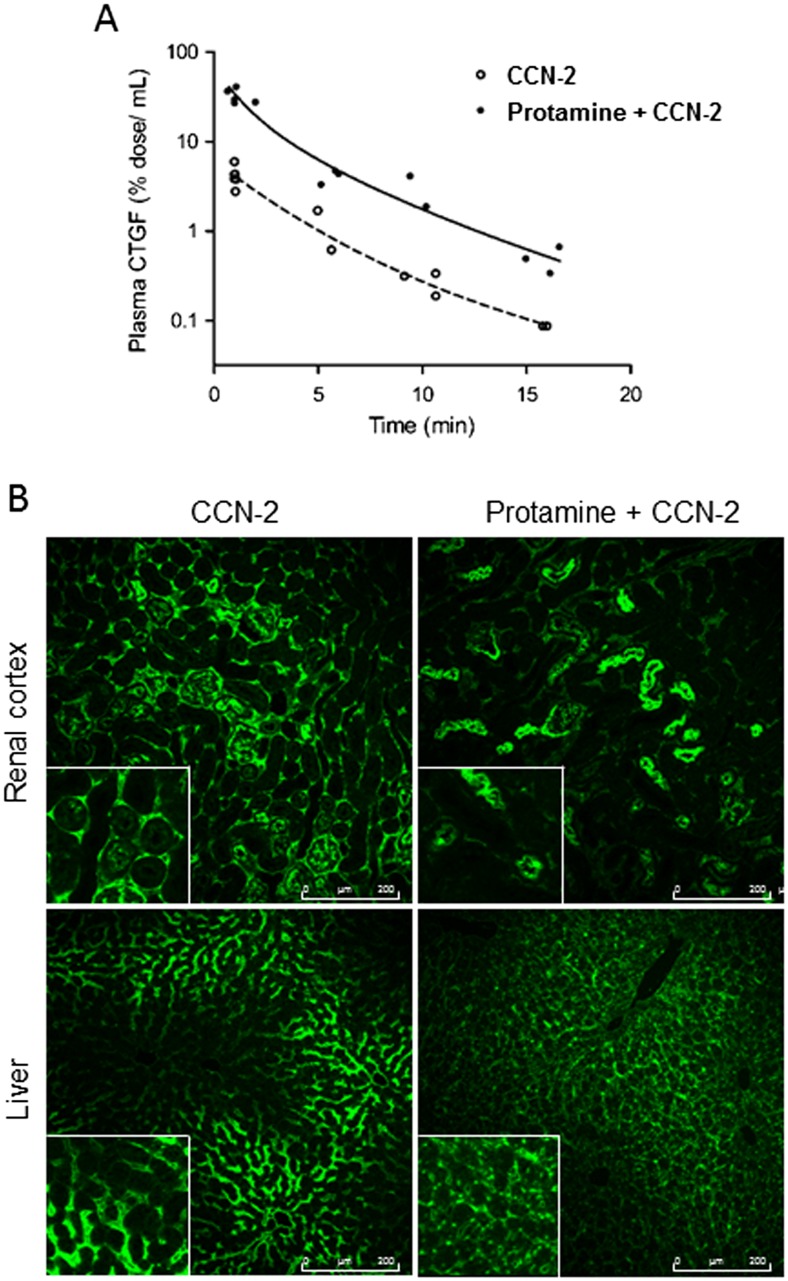
**a**-**b** Pharmacokinetic profile after single intravenous dose of recombinant human CCN-2 preceded by injection of an excess of protamine in mice. **a** Plasma disappearance curve of CCN-2. Administration of protamine prior to CCN-2 reduced distribution volume but not elimination rate. Plasma concentration is expressed as percentage of injected dose per mL plasma. Each dot represents one animal. **b** Immunofluorescence of kidney and liver, 5 min after CCN-2 administration, by using human anti- CCN-2 (*green*), visualized by confocal laser scanning microscopy. Protamine prior to CCN-2 (*right*) reduces interstitial staining

To examine how binding of HSPGs in vivo influenced the pharmacokinetic profile of plasma CCN-2, we performed clearance studies in the presence of an excess of protamine, which prevents HSPGs from interacting with their ligands (Warshawsky et al. 1996; Narita et al. 1995). Protamine administered as a single dose 1 min prior to CCN-2 injection resulted in about 7-fold higher plasma CCN-2 levels at all time points as compared to single CCN-2 bolus injection (*p* < 0.05, Fig. 3a) and in a decrease of interstitial staining 5 min after bolus injection, in particular in the kidney (Fig. 3b). The parallel shift of the plasma disappearance curve indicates that the distribution volume of CCN-2 was reduced, while clearance remained unaffected by protamine. Thus, binding to HSPGs appears to be involved in distribution of full length CCN-2 but not in its elimination from the circulation.

## Discussion

The main finding of our studies is that circulating full length CCN-2 is primarily cleared by fast hepatic metabolism via a RAP-sensitive pathway, most likely involving LRP1.

Full length CCN-2 distributed mainly to the liver and was taken up by hepatocytes. The half-life of CCN-2 was markedly prolonged and hepatic uptake substantially reduced after administration of RAP, which suggests that uptake in hepatocytes is mediated by an LDLR family protein. This is in accordance with previous in vitro cell binding experiments, showing that CCN-2 is an LRP1 ligand (Segarini et al. 2001; Gao and Brigstock 2003). We confirmed this by surface plasmon resonance analysis. Rapid disappearance of all intracellular inclusions in hepatocytes 30 min after bolus injection in mice suggests fast degradation after endocytosis, in line with rapid LRP1-mediated internalization and subsequent intracellular degradation as observed in vitro (Segarini et al. 2001). LRP1 is responsible for plasma clearance of various proteins including coagulation factors and lipoprotein remnants (Lillis et al. 2008). LRP1 is most prominent in liver, but is also present in various other tissues, including lung, central nervous system and lymphoid tissues (Moestrup et al. 1992). Interestingly, mice in which the LRP1 gene is deleted in smooth muscle cells developed aortic dilatation and medial thickening that is accompanied by local accumulation of the CCN-2 protein, indicating that LRP1 maintains the vasculature integrity by modulation of CCN-2 protein levels (Muratoglu et al. 2013). We did not observe evident CCN-2 accumulation in vessels, lung, heart or spleen. Inhibition of LRP1 binding by RAP has been applied before to prove LRP1-involvement in clearance of various LRP1 ligands in vivo (Narita et al. 1995; Saenko et al. 1999; Sarafanov et al. 2001; Schwarz et al. 2000). In addition to LRP1, RAP is known to block ligand binding to other endocytic cell surface receptors of the LDL receptor family members (Bovenschen et al. 2003).

Previous reports found that CCN-2 binds LRP1 via domain 3, since blocking of LRP1 reduced binding of full length CCN-2 and the C-terminal half, but not binding of domain 4 alone, to activated hepatic stellate cells (Gao and Brigstock 2003). Surface plasmon resonance experiments now confirm that CCN-2 is indeed binding via an epitope on its C-terminal half to LRP1, in agreement with previous pharmacokinetic studies with the N-terminal fragment of CCN-2, that did not show any hepatic uptake in vivo (Gerritsen et al. 2010).

Thus far, elevated plasma levels of full length CCN-2 in chronic liver disease have been considered to be secondary to increased production in fibrotic liver tissue (Gressner et al. 2006; Guo-Qiu et al. 2010). Increased platelet activation in advanced liver disease (Panasiuk et al. 2001) might also contribute to elevated CCN-2, since platelets contain large amounts of full length CCN-2 that is released upon activation (Cicha et al. 2004; Miyazaki et al. 2010). We here show that hepatic metabolism is the main elimination route of full length CCN-2. Hence reduced elimination might also contribute to accumulation of full length CCN-2 in advanced liver disease (Kovalenko et al. 2009; Zhang et al. 2009; Guo-Qiu et al. 2010). Hollestelle et al. showed that liver cirrhosis is associated with decreased expression of LRP1 (Hollestelle et al. 2004). However, additional experiments, e.g. elimination studies in experimental liver cirrhosis, would be needed to explore the postulated contribution of reduced hepatic removal to elevated CCN-2 levels in chronic liver disease.

In mice we found a relatively large initial distribution volume exceeding the extracellular fluid volume which is remarkable for a hydrophilic glycosylated protein of approximately 37 kD and suggests tissue or plasma protein binding. Previous studies reported in vitro binding of CCN-2 to HSPGs (Gao and Brigstock 2004), which are widely distributed in all tissues. The substantial decrease in distribution volume of CCN-2 after administration of protamine suggests that CCN-2 also binds to HSPGs in vivo. This is supported by the finding that interstitial staining in the kidney was markedly reduced after protamine administration, although in the liver this was less evident, possibly as a consequence of CCN-2 binding to other molecules, including LRP1. In vitro*,* CCN-2 binds to HSPGs via domain 4 on its C-terminal half (Gao and Brigstock 2004). Consistently, interstitial staining in the kidney was virtually absent after in vivo administration of the N-terminal fragment (Gerritsen et al. 2010). Binding to HSPGs has been shown to facilitate LRP1 mediated degradation of the LRP1 ligands factor VIII and tissue activator pathway inhibitor (Sarafanov et al. 2001; Narita et al. 1995; Lillis et al. 2008). However, for CCN-2 we observed no increase of plasma half-life upon blocking its binding to HSPGs by protamine, suggesting that in vivo CCN-2 degradation by LRP1 is not facilitated by binding to HSPGs.

We used antibodies detecting both full length CCN-2 and the N-terminal fragment for immunohistochemistry and ELISA. Although experiments were of short duration, proteolytic cleavage of the full length molecule into its N- and C-terminal halves could theoretically have influenced the results. This seems unlikely, since in similar experiments with the N-fragment, we observed a much smaller distribution volume, efficient renal clearance and no hepatic uptake at all (Gerritsen et al. 2012). Although we cannot exclude that the small percentage of renal clearance observed upon infusion studies of full length CCN-2 in rats was partly due to N-fragment clearance, some glomerular sieving is also not unlikely for the full length protein that has a diameter of 36–38 kDa.

In conclusion, our findings show that full length CCN-2 is primarily eliminated by the liver via a fast RAP-sensitive, probably LRP1-mediated, pathway. The large initial distribution volume of full length CCN-2 is due to a protamine-sensitive process, presumably CCN-2 binding to HSPGs, but we have no indication that CCN-2 interaction with HSPGs facilitates RAP-sensitive hepatic catabolism.

## Abbreviations

CCN: CTGF/CYR61/NOV
CKD: chronic kidney disease
CTGF: connective tissue growth factor
CYR61: cysteine-rich 61
*E. coli*: *Escherichia coli*
ELISA: enzyme-linked immunosorbent assay
ERPF: effective renal plasma flow
GFR: glomerular filtration rate
GST-RAP: glutathione S-transferase-fused receptor-associated protein
HSPG: heparan sulphate proteoglycan
LDL: low density lipoprotein
LRP: low density lipoprotein receptor-related protein
NOV: nephroblastoma overexpressed
PAH: para-aminohippuric acid
RAP: receptor-associated protein
SE: standard error
SEM: standard error of the mean
$\overset{\bullet }{V}$: urinary flow rate
